# The Surprise Question and Health-Related Quality of Life in Patients on Hemodialysis: A Cross-Sectional Multicenter Study

**DOI:** 10.1089/pmr.2023.0093

**Published:** 2024-08-02

**Authors:** Jeanette M. Wallin, Stefan H. Jacobson, Lena Axelsson, Jenny Lindberg, Carina I. Persson, Jenny Stenberg, Agneta Wennman-Larsen

**Affiliations:** ^1^ Department of Nursing Science, Sophiahemmet University, Stockholm, Sweden.; ^2^ Division of Nephrology, Department of Clinical Sciences, Karolinska Institutet, Danderyd Hospital, Stockholm, Sweden.; ^3^ Department of Clinical Sciences, Unit of Medical Ethics, Lund University, Lund, Sweden.; ^4^ Department of Research, Region Kalmar County, Kalmar, Sweden.; ^5^ Department of Medical Sciences, Uppsala University, Uppsala, Sweden.; ^6^ Department of Clinical Neuroscience, Karolinska Institutet, Stockholm, Sweden.

**Keywords:** communication, health-related quality of life, hemodialysis, palliative care, Surprise Question

## Abstract

**Background:**

The Surprise Question (SQ) is a common method aimed at identifying frail patients who need serious illness conversations to integrate a palliative approach. However, little is known about whether the SQ identifies patients on hemodialysis who perceive that they are declining or have low health-related quality of life (HRQoL)—important aspects when considering the need for serious illness conversations.

**Objective:**

To explore how nurses and physicians’ responses to the SQ are associated with patients’ self-reported HRQoL.

**Design:**

Cross-sectional study.

**Subjects:**

In total, 282 patients on hemodialysis were included.

**Measurements:**

One nurse and one physician responded to the SQ for each patient. The patient-reported HRQoL was measured with the RAND 36-Item Health Survey 1.0 (RAND-36) and the EuroQual vertical visual analogue scale (EQ-VAS) from the EuroQual-5 Dimension Questionnaire (EQ-5D).

**Results:**

Nurses’ responses “no, not surprised” to the SQ were associated with patient-reported worsened health compared to one year ago (RAND-36), and lower perceived overall health (EQ-VAS). Physicians’ responses “no, not surprised” were associated with lower overall health and lower physical functioning. Patient-reported pain, general health, fatigue, and emotional and social aspects were not associated with responses to the SQ.

**Conclusions:**

The findings indicate that the SQ identifies patients on hemodialysis who report low overall health and low physical functioning. However, the SQ did not identify patients who reported pain, emotional problems, or fatigue, which are also important aspects to consider in identifying needs for serious illness conversations, symptom management, and to be able to integrate a palliative approach.

## Background

Hemodialysis for patients with kidney failure is life-prolonging. Most patients are elderly and frail^1^ and often experience several symptoms and comorbidities^2,3^ with impacts on health-related quality of life (HRQoL).^4–6^ This implies that many of these patients would benefit from integrating the hemodialysis care with a palliative approach. To be able to integrate a palliative approach for these patients, serious illness conversations including end-of-life issues are fundamental. Having these conversations in advance has been shown to lead to care that is more consistent with patients’ preferences, including preferred place of death.^7^ However, serious illness conversations do not occur as often as they should.^8,9^ Studies^9,10^ show that health care professionals are hesitant to initiate conversations, preferring to wait for signals from the patient owing to the fear of upsetting them.^9^ On the other hand, it has been shown that the patients and significant others are thinking of death and dying and want to talk about issues related to end of life including withdrawal of hemodialysis, especially when patients’ health is deteriorating or when their quality of life is low.^11,12^ Nevertheless, patients may expect the health care professionals to initiate these conversations.^11–13^ In addition, it has been shown that the successive decline of the patients’ illness is disregarded by physicians and nurses,^9,14^ which may be another reason why patients approaching the end of life and in need of serious illness conversations are not identified.

Several studies have described different ways to help health care professionals identify timepoints on when to initiate conversations in serious illness.^15^ The Surprise Question (SQ) “Would I be surprised if this patient died within the next 6 (or 12) months?,” as a single question or together with other parameters, has been shown to be a common method to identify patients in need of serious illness conversations.^15–17^ In a hemodialysis context, the SQ has been shown to significantly predict short-term survival^18–20^ but with lower accuracy and sensitivity than in oncology.^21^ The responses also differ between nurses and physicians’ ratings^20^ and regarding specific patients.^22^ Although the SQ has been evaluated for predicting mortality,^18–20,23,24^ the SQ was originally developed as an intuitive question to identify patients who are frail but who will not necessarily die within a specific timeframe.^25^ To be able to identify all patients who are in need of serious illness conversations, it is important to understand whether health care professionals’ responses to the SQ actually capture patients who perceive that they are declining or have low HRQoL. This is because these patients may have thoughts concerning death and dying, and of issues related to hemodialysis withdrawal. Both health care professionals’ judgment and patients’ experiences are important in identifying the need for serious illness conversations. This comparison may also be important in evaluating the usefulness of the SQ, as not all patients are able or willing to respond to self-reported HRQoL measures.

To the best of our knowledge, comparing the SQ together with dimensions of HRQoL in patients treated with hemodialysis has only been evaluated in one previous study,^19^ and no study has evaluated how nurses and physicians’ responses to the SQ are associated with specific dimensions of self-reported HRQoL in this group. Therefore, the aim was to explore how nurses and physicians’ responses to the SQ are associated with patients’ self-reported HRQoL.

## Material and Methods

In this cross-sectional study, data were collected from patients, nurses, physicians, and from the Swedish Renal Registry. The data collection took place between January 2020 and June 2022 and included nine hemodialysis in-centers located in three Swedish regions. Hemodialysis nurses in Sweden are registered nurses who usually have the primary nursing responsibility for three to four patients and are also responsible for conducting the hemodialysis procedure. Physicians with the primary responsibility for medical care at the dialysis center are most often specialized nephrologists.

The eligibility criteria were patients ≥18 years, treated with in-center hemodialysis for ≥3 months. Information and forms regarding informed consent were available in the six most spoken languages of patients at these facilities: Swedish, English, Arabic, Polish, Bosnian-Croatian-Serbian, and Somali. This study is part of a multicenter prospective project aiming to develop and evaluate an instrument to support the initiation of serious illness conversations for patients on hemodialysis. From the overall project, one previous study has been published.^22^

### Variables and data sources

In the present study, patients were invited to complete a questionnaire including the RAND 36-Item Health Survey 1.0 (RAND-36),^26^ the EuroQual-5 Dimension Questionnaire (EQ-5D),^27^ and demographic data.

The RAND-36 was chosen since it is recommended for annual follow-up of patients on hemodialysis in the Swedish Renal Registry.^3^ It is also a widely used generic questionnaire developed from the Medical Outcome Study (MOS) to measure HRQoL.^26^ There are two versions, RAND-36 that is free of charge and SF-36 that requires license payment. RAND-36 and SF-36 are comparable with each other.^26^ RAND-36 consists of 36 questions of which 35 are divided into eight sub-scales, namely, physical functioning, role-functioning/physical, pain, general health, energy/fatigue, social functioning, role-functioning/emotional, and emotional well-being, and one single item, that is, health change, measuring change in health during the past 12 months.^26,28^ The score of each domain and the single question on health change is transformed into a 0–100 point scale, with a higher score indicating higher perceived health status.^26^ The response option for health change ranges from “much worse now than one year ago” (0) to “much better now than one year ago” (100).^26^

The EQ-5D was chosen since it is available in many languages, making it possible to include groups of patients not speaking Swedish, and it is also a widely used generic questionnaire.^29^ For this study, one of the six questions in EQ-5D, the EQ vertical visual analogue scale (EQ-VAS) measuring self-rated health, was used. The response option for EQ-VAS ranges from “the worst health you can imagine” (0) to “the best health you can imagine” (100).^27^

The SQ^25^ was responded to by physicians and nurses regarding 6- and 12-month timeframes, i.e., “Would I be surprised if this patient died within 6 months?” and “Would I be surprised if this patient died within 12 months?” The response options were “Yes, I would be surprised” or” No, I would not be surprised.” In the results, the responses are coded as, “yes, surprised” and “no, not surprised.”

Comorbidities were registered by the physicians using the Charlson Comorbidity Index (CCI).^30^ In this study, a modified version of the CCI was used in which lymphoma and leukemia are included in “any malignancy.” The score for this modified version ranges from 0 to 29 with each comorbidity being weighted. CCI with higher scores indicates a higher comorbidity burden.

Patients’ physical capability was assessed by the nurses using the Eastern Cooperative Oncology Group/World Health Organization Performance Status Scale (ECOG/WHO).^31^ Performance status (ECOG/WHO) response options range from 0—fully active to 4—completely disabled.^31^

Age, gender, information about hemodialysis procedure, and laboratory findings were obtained from the Swedish Renal Registry.^3^

### Study procedure

Both physicians and nurses received an individual list of patients who met the inclusion criteria and in whose care they were primarily involved. Each patient was evaluated by one nurse and one physician, responding to the SQ. To avoid bias in response to the SQ, nurses and physicians were instructed to answer the SQ before registering performance status and CCI, respectively.

### Statistical methods

Descriptive statistics are used to present the characteristics of patients. Mean values and standard deviations (SD) are used for normally distributed continuous data, and for nonnormally distributed data, median and interquartile range (IQR) are presented.

Using logistic regression, odds ratios (OR) with 95% confidence intervals (CI) and significance set at*p* < 0.05 were calculated to assess the association between nurses and physicians’ responses to the SQ regarding the 6- and 12-month timeframes and patients’ self-reported HRQoL, respectively. All statistical analyses were performed using SPSS software, version 27.

### Ethical considerations

The study was approved by the Swedish Ethical Review Authority, Dnr: 2019–03877. All included patients had given their written informed consent to participate. Regarding data from the Swedish Renal Registry, the law in Sweden permits data from health care quality registries to be used in research. Patients are entitled to opt-out, but no separate patient informed consent for individual studies is required.

## Results

**FIG. 1.**
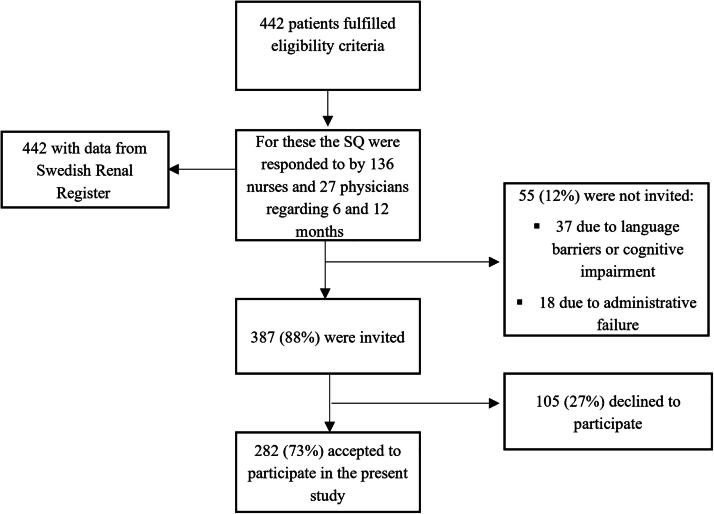
Inclusion process of patients for the project.

Registry data were collected from 442 patients for whom 136 nurses and 27 physicians responded to the SQ, respectively, regarding 6- and 12-month timeframes (Fig. 1). Of these 442 patients, 387 were invited to provide informed consent for answering the HRQoL questionnaires. Fifty-five (12%) patients were not invited owing to administrative failure, language barriers, or cognitive impairment disabling the provision of informed consent. Of the 387 patients, 282 (73%) patients agreed to participate, whereas 105 declined (Fig. 1). For these 282 patients, the SQ was responded to by 113 nurses and 25 physicians. In the final sample, it was discovered that three of the included patients had been treated with hemodialysis for less than three months but more than two months, and they were included in the analyses.

In a drop-out analysis comparing the 282 included patients to the remaining 160, of whom 55 were excluded in the prescreening process and 105 declined to participate, it was found that the enrolled patients were significantly older—68 ± 13 years—compared with the 160 nonparticipating patients—64 ± 16.2 years (*p* = 0.017). There were no significant differences between participants and nonparticipants regarding performance status, sex, comorbidities, albumin, hemoglobin, and time since the start of kidney replacement therapy.

### Patient demographics and clinical characteristics

Of the 282 patients included, 64% were male, and the mean age was 68 years (Table 1). A majority of the patients were born in Sweden (67%), whereas 14% were born in another European country and 16% were born outside Europe. Most of the patients (57%) had an education level of ≥10 years, whereas 38% had a level of education of <10 years (Table 1). According to patient performance status, 28% were fully active (score 0), whereas 20% were either limited in self-care or completely disabled (score 3–4). The most common comorbidities were diabetes (47%), followed by congestive heart failure (32%) and myocardial infarction (26%) (Table 1).

**Table 1.** Patient Demographics, Performance Status, Comorbidities, and Clinical Characteristics (*n* = 282)

Variable	n = 282
Sex	
Male	63.8% *n* = 180
Female	36.2% *n* = 102
Age, year	67.7 ± 13.1
Country of birth	
Sweden	67.4% *n* = 190
Another Nordic country	5.7% *n* = 16
Another European country	7.8% *n* = 22
Outside Europe	15.6% *n* = 44
Missing	3.5% *n* = 10
Marital status	
Living alone	37.2% *n* = 105
Married/Partner/Other	59.2% *n* = 167
Missing	3.5% *n* = 10
Education	
Non	0.7% *n* = 2
Elementary school, 1–9 years	37.2% *n* = 105
Grammar/Secondary school, 10–12 years	32.6% *n* = 92
College or university ≥13 years	24.1% *n* = 68
Missing	5.3% *n* = 15
Number of hospitalizations during the past six month (self-reported)	
0–1 time	64.5% *n* = 182
2–3 times	24.1% *n* = 68
4– times	7.4% *n* = 21
Missing	3.9% *n* = 11
ECOG/WHO performance status;	
0	27.7% *n* = 78
1	18.8% *n* = 53
2	27.7% *n* = 78
3	15.6% *n* = 44
4	3.9% *n* = 11
Missing	6.4% *n* = 18
Comorbidities (+)	
Diabetes	46.5% *n* = 131
Congestive heart failure	32.3% *n* = 91
Myocardial infarction	26.2% *n* = 74
Peripheral vascular disease	24.1% *n* = 68
Charlson Comorbidity Index (CCI)	5 ± 2.3
Time with kidney replacement therapy (months)	38.0 (16.0–78.8)
Vascular access	
Fistula	46.1% *n* = 130
Graft	14.5% *n* = 41
CVC	36.5% *n* = 103
Missing	2.8% *n* = 8
Laboratory test results	
stdKt/V	2.3 ± 0.4
Albumin (g/l)	32.1 ± 4.3
Hemoglobin (g/l)	113.1 ± 13.4

Mean and standard deviation (±) are reported for normally distributed values. Median and interquartile range (IQR) and frequencies as percentages (%) and numbers (*n*) are reported for nonparametric values.

ECOG/WHO performance status: 0—Fully active, able to carry on all predisease performance without restriction; 1—Restricted in physically strenuous activity but ambulatory and able to carry out work of a light or sedentary nature, e.g., light housework office work; 2—Ambulatory and capable of all self-care but unable to carry out any work activities; up and about more than 50% of waking hours; 3—Capable of only limited self-care; confined to bed or chair more than 50% of waking hours; 4—Completely disabled; cannot carry on any self-care; totally confined to bed or chair.

CVC, central venous catheter; ECOG, The Eastern Cooperative Oncology Group; WHO, World Health Organization.

### Patients’ self-reported HRQoL

**FIG. 2.**
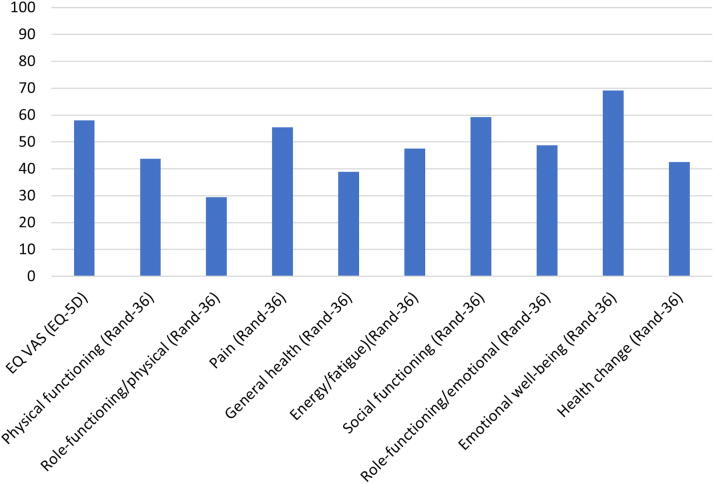
The study population’s mean of self-reported health-related quality of life.

The mean value for EQ-VAS was 58 ± 21.1. For RAND-36, the mean values for the various domains were physical functioning 43.7 ± 29.4, role functioning/physical 29.4 ± 36.5, pain 55.5 ± 29.3, general health 38.9 ± 21.3, energy/fatigue 47.6 ± 22.1, social functioning 59.3 ± 28.6, role functioning/emotional 48.7 ± 42.5, emotional well-being 69.2 ± 21.0, and health change 42.5 ± 26.4 (Fig. 2).

### Associations between nurses’ responses to the SQ and patients’ self-reported HRQoL

**FIG. 3.**
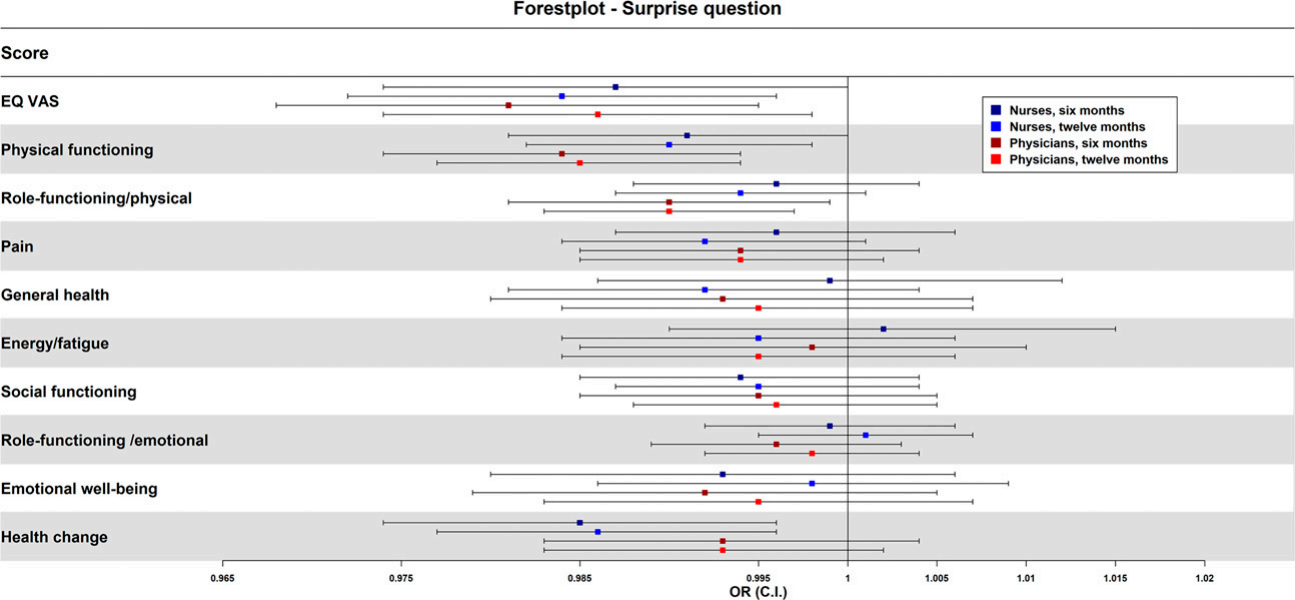
Associations between patients’ self-reported health-related quality of life and nurses and physicians’ responding “no, not surprised” to the Surprise Question regarding 6- and 12 months using *unadjusted logistical regression analysis* to calculate odds ratios with 95% confidence intervals.

In the unadjusted multivariable regression analyses, nurses’ responses “no, not surprised” to the SQ regarding six months were associated with lower score in health change, i.e., worsened health than one year ago (OR = 0.985, *p* = 0.007). Regarding 12 months, nurses’ responses “no, not surprised” were associated with lower EQ-VAS (OR = 0.984, *p* = 0.008), lower physical functioning (0.990 *p* = 0.020), and lower score in health change (OR = 0.986, *p* = 0.005) (Table 2, Fig. 3). There were no associations between role-functioning/physical, pain, general health, energy/fatigue, role-functioning/emotional, social functioning, or emotional well-being and nurses’ responses to the SQ (Table 2, Fig. 3).

**Table 2.** Associations Between Patients’ Self-Reported Health-Related Quality of Life (EuroQual vertical Visual analogue Scale in EuroQual-5 Dimension Questionnaire and RAND 36-Item Health Survey 1.0) and Nurses and Physicians’ Responses to the Surprise Question—“Would I Be Surprised If This Patient Died Within Six Months” and “Would You Be Surprised If This Patient Died Within 12 Months” Using Unadjusted and Adjusted Logistical Regression Analysis to Calculate Odds Ratios (OR), 95% Confidence Intervals (CI), and Significance Level (*p*).

	Unadjusted		Model 1^a^		Model 2^b^	
	OR (95% CI)	Sig.	OR (95% CI)	Sig.	OR (95% CI)	Sig.
Nurses’ responses—“Would I be surprised if this patient died within the next six months?”						
EQ-5D						
EQ-VAS	0.987 (0.974–1.000)	0.057	0.989 (0.976–1.003)	0.115		
Rand-36						
Physical functioning	0.991 (0.981–1.000)	0.059	0.994 (0.985–1.004)	0.270		
Role functioning/physical	0.996 (0.988–1.004)	0.374				
Pain	0.996 (0.987–1.006)	0.434				
General health	0.999 (0.986–1.012)	0.842				
Energy/Fatigue	1.002 (0.990–1.015)	0.742				
Social functioning	0.994 (0.985–1.004)	0.243				
Role functioning/emotional	0.999 (0.992–1.006)	0.747				
Emotional well-being	0.993 (0.980–1.006)	0.275				
Health change	0.985 (0.974–0.996)	0.007*	0.986 (0.986–0.998)	0.019*	0.986 (0.974–0.998)	0.021*
Nurses’ responses—“Would I be surprised if this patient died within the next 12 months?”						
EQ-5D						
EQ-VAS	0.984 (0.972–0.996)	0.008*	0.986 (0.973–0.998)	0.024*	0.985 (0.973–0.998)	0.021*
Rand-36						
Physical functioning	0.990 (0.982–0.998)	0.020*	0.994 (0.985–1.003)	0.166		
Role functioning/physical	0.994 (0.987–1.001)	0.074	0.995 (0.988–1.003)	0.214		
Pain	0.992 (0.984–1.001)	0.080	0.994 (0.985–1.002)	0.158		
General health	0.992 (0.981–1.004)	0.193				
Energy/Fatigue	0.995 (0.984–1.006)	0.333				
Social functioning	0.995 (0.987–1.004)	0.259				
Role functioning/emotional	1.001 (0.995–1.007)	0.815				
Emotional well-being	0.998 (0.986–1.009)	0.696				
Health change	0.986 (0.977–0.996)	0.005*	0.988 (0.978–0.998)	0.019*	0.988 (0.987–0.998)	0.019*
Physicians’ responses to—“Would I be surprised if this patient died within the next six months?”						
EQ-5D						
EQ-VAS	0.981 (0.968–0.995)	0.006*	0.982 (0.968–0.997)	0.015*	0.982 (0.986–0.997)	0.017*
Rand-36						
Physical functioning	0.984 (0.974–0.994)	0.002*	0.988 (0.977–0.998)	0.022*	0.988 (0.977–0.999)	0.027*
Role functioning/physical	0.990 (0.981–0.999)	0.023*	0.992 (0.983–1.001)	0.080	0.992 (0.983–1.001)	0.090
Pain	0.994 (0.985–1.004)	0.229				
General health	0.993 (0.980–1.007)	0.334				
Energy/Fatigue	0.998 (0.985–1.010)	0.716				
Social functioning	0.995 (0.985–1.005)	0.315				
Role functioning/emotional	0.996 (0.989–1.003)	0.267				
Emotional well-being	0.992 (0.979–1.005)	0.240				
Health change	0.993 (0.983–1.004)	0.233				
Physicians’ responses to—“Would I be surprised if this patient died within the next 12 months?”						
EQ-5D						
EQ-VAS	0.986 (0.974–0.998)	0.018*	0.987 (0.975–1.000)	0.046*	0.987 (0.974–0.999)	0.038*
Rand-36						
Physical functioning	0.985 (0.977–0.994)	0.001*	0.989 (0.980–0.998)	0.019*	0.989 (0.979–0.998)	0.018*
Role functioning/physical	0.990 (0.983–0.997)	0.008*	0.992 (0.985–1.000)	0.044*	0.992 (0.985–1.000)	0.047*
Pain	0.994 (0.985–1.002)	0.149				
General health	0.995 (0.984–1.007)	0.444				
Energy/Fatigue	0.995 (0.984–1.006)	0.369				
Social functioning	0.996 (0.988–1.005)	0.378				
Role functioning/emotional	0.998 (0.992–1.004)	0.483				
Emotional well-being	0.995 (0.983–1.007)	0.388				
Health change	0.993 (0.083–1.002)	0.122				

The Reference for the Outcome is “Yes, Surprised.”

^a^ Model 1 adjusted for age.

^b^ Model 2 adjusted for age, sex, and time with kidney replacement therapy.

*p < 0.05.

CI, confidence interval; OR, odds ratio.

In the multivariable regression analyses adjusted for patient age (Model 1), a lower score in health change was associated with nurses’ responses “no, not surprised” regarding six months. Regarding 12 months, lower EQ-VAS and lower score in health change were associated with nurses’ responses “no, not surprised” (Table 2). These associations remained in Model 2 when adjusted for patient age, sex, and time with kidney replacement therapy (Table 2).

### Associations between physicians’ responses to the SQ and patients’ self-reported HRQoL

In the unadjusted multivariable regression analyses regarding 6 and 12 months, physicians’ responses “no, not surprised” to the SQ were associated with lower EQ-VAS (OR = 0.981/0.986, *p* = 0.006/0.018), lower physical functioning (OR = 0.984/0.985, *p* = 0.002/0.001), and lower role functioning/physical (OR = 0.990/0.990, *p* = 0.023/0.008) (Table 2, Fig. 3). There were no associations between pain, general health, energy/fatigue, social functioning, role-functioning/emotional, emotional well-being, or health change and physicians’ responses to the SQ (Table 2, Fig. 3).

In the multivariable regression analyses adjusted for patient age (Model 1), lower EQ-VAS and lower physical functioning were associated with physicians’ responses “no, not surprised” regarding six months, whereas lower EQ-VAS and lower physical functioning and lower role functioning/physical were associated with responding “no, not surprised” regarding 12 months (Table 2). These associations remained in Model 2 when adjusted for patient age, sex, and time with kidney replacement therapy (Table 2).

## Discussion

The present study aimed to explore how nurses and physicians’ responses to the SQ are associated with patients’ self-reported HRQoL. Regarding six months, nurses’ responses “no, not surprised” were associated with lower score in health change, i.e., worsened health than one year ago, and physicians’ responses were associated with lower EQ-VAS and physical functioning. Regarding 12 months, nurses’ responses were associated with lower score in health change and lower EQ-VAS, and physicians’ responses to the SQ were associated with lower EQ-VAS, lower physical functioning, and lower role functioning/physical. No other dimensions of HRQoL were associated with nurses or physicians’ responses to the SQ.

The association between nurses and physicians’ responses to the SQ and patients’ self-reported health measured by EQ-VAS, health change, and the physical functioning of RAND-36 show that the SQ may identify patients with lower perceived health status and lower physical functioning. Both nurses and physicians have a central role in identifying patients in need of serious illness conversations and the findings from this study indicate that nurses and physicians’ responses to the SQ have the potential to identify patients who may be in need of such conversations. This is in line with the original aim of SQ that was not actually developed as a predictor for mortality but rather to be used as an intuitive question for health care professionals to identify patients who are frail but who will not necessarily die within a specific timeframe.^25^ Even if the original aim of the SQ was not to predict mortality, it has mostly been evaluated regarding mortality.^21,23,32^ The accuracy of the SQ as a measure of mortality in this group has however been problematized in previous studies.^21,23,32^ Compared to the SQ as a single question, a model where the SQ together with risk factors for patients on hemodialysis such as older age, lower albumin, dementia, and peripheral vascular disease is better to identify patients at high risk for short-term mortality.^18,23^ However, further research is needed to understand whether the SQ along with patients’ self-reported HRQoL can also increase the SQ accuracy for the short-term mortality rate.

The identified association between nurses and physicians’ responses to the SQ and patient self-reported HRQoL is also important since it shows that the SQ may identify patients who experience deteriorating health, which in a previous study has been shown to raise thoughts around death and a desire for health care professionals to initiate conversations.^11^ Patients’ self-reported decline in physical functioning has also previously been shown to be an early sign of frailty, especially regarding physical functioning in RAND-36.^33^ Physical functioning in RAND-36 is associated with objective measures of physical decline such as slower gait speed and lower handgrip strength,^33^ which underpins the importance of the SQ associations to HRQoL. A previous study has also shown that identifying patients in need of serious illness conversations is a complex process^34^ and health care professionals find it important but difficult to identify patients’ readiness for these conversations.^9^ We can only speculate but the results from the present study might indicate that the responses to the SQ also can identify patients who are in need of and who may be ready for these conversations since they experience a decline in health or in HRQoL. However, this interpretation of an association between the SQ and the patient’s readiness has to be done with caution and needs to be further investigated.

Even if the original purpose of the SQ was not to identify patients with certain symptoms, such as pain, fatigue, and emotional problems,^25^ from a palliative approach it is notable that nurses and physicians’ responses to the SQ did not seem to capture these aspects. Thus, it is known that in the last year of life the experiences of symptoms increase for patients on hemodialysis and their physical and mental quality of life decrease.^35^ Our results are also consistent with a previous study showing no association between patients’ perceived pain and nurse practitioners’ responses to the SQ.^19^ Thus, it seems that the SQ does not facilitate the identification of patients with pain, emotional problems, or fatigue. These are highly relevant symptoms and aspects to consider in identifying needs of serious illness conversations to be able to achieve the best possible HRQoL when the patient is approaching the end of life.

### Strengths and limitations

A strength of this study is the high response rate in this vulnerable group, the inclusion of patients from several facilities in three different Swedish regions, and that one-third of the included patients were born outside Sweden. In Sweden, hemodialysis care is covered by national health insurance and is not dependent on a patient’s income, which thus avoids selection effects based on socioeconomic status. A limitation is however that those who participated were older than the nonparticipants, which may limit the generalization of the results for younger age groups. A strength is also that each patient was evaluated with the SQ by one nurse and one physician, both involved in the specific patient’s care and familiar to their illness trajectory. In other studies, the SQ has been responded to by one or a few physicians and/or nurses for all patients at one dialysis unit.^21^ A limitation is that the nurses and physicians responded to the SQ regarding both 6- and 12-month timeframes, which may have led to these responses being affected by each other.

## Conclusions

The findings indicate that the SQ identifies patients on hemodialysis who report low overall health and low physical functioning. However, the SQ did not identify patients who reported pain, emotional problems, or fatigue, which are also important aspects to consider in identifying needs for serious illness conversations, symptom management, and to be able to integrate a palliative approach.

## Notes

### Abbreviations Used

CCI: Charlson Comorbidity Index
ECOG: The Eastern Cooperative Oncology Group
EQ-5D: EuroQual-5 Dimension Questionnaire
EQVAS: EuroQual vertical visual analogue scale
HRQoL: Health-Related Quality of Life
Rand-36: RAND 36-Item Health Survey 1.0
stdKt/V: The parameter std Kt/V is a measurement of dialysis efficacy. Standardized Kt/V; K – dialyzer clearance of urea, t – dialysis time, V – volume of distribution
The SQ: The Surprise Question
WHO: World Health Organization
